# A Multisensor Data Fusion Approach for Predicting Consumer Acceptance of Food Products

**DOI:** 10.3390/foods9060774

**Published:** 2020-06-11

**Authors:** Víctor M. Álvarez-Pato, Claudia N. Sánchez, Julieta Domínguez-Soberanes, David E. Méndoza-Pérez, Ramiro Velázquez

**Affiliations:** 1 Facultad de Ingeniería, Universidad Panamericana, Aguascalientes 20290, Mexico; valvarez@up.edu.mx (V.M.Á.-P.); cnsanchez@up.edu.mx (C.N.S.); 2 Escuela de Negocios Gastronómicos, Universidad Panamericana, Aguascalientes 20290, Mexico; jdominguez@up.edu.mx (J.D.-S.); demendoza@up.edu.mx (D.E.M.-P.)

**Keywords:** consumer acceptance prediction, data fusion, emotion recognition, facial expression recognition, galvanic skin response, machine learning, neural networks, sensory analysis

## Abstract

Sensory experiences play an important role in consumer response, purchase decision, and fidelity towards food products. Consumer studies when launching new food products must incorporate physiological response assessment to be more precise and, thus, increase their chances of success in the market. This paper introduces a novel sensory analysis system that incorporates facial emotion recognition (FER), galvanic skin response (GSR), and cardiac pulse to determine consumer acceptance of food samples. Taste and smell experiments were conducted with 120 participants recording facial images, biometric signals, and reported liking when trying a set of pleasant and unpleasant flavors and odors. Data fusion and analysis by machine learning models allow predicting the acceptance elicited by the samples. Results confirm that FER alone is not sufficient to determine consumers’ acceptance. However, when combined with GSR and, to a lesser extent, with pulse signals, acceptance prediction can be improved. This research targets predicting consumer’s acceptance without the continuous use of liking scores. In addition, the findings of this work may be used to explore the relationships between facial expressions and physiological reactions for non-rational decision-making when interacting with new food products.

## 1. Introduction

Consumer response and purchase decision are always uncertain and changing. Nevertheless, there is a consensus that consumer behavior has both psychological and physiological components that have a great influence on consumer choices [1,2].

Among the variety of psychological aspects of consumer behavior, we can find the affective response or the different feelings that a purchase may induce in the buyer: uniqueness, proudness associated with social status, excitement, sense of responsibility, and confidence, among others [3]. The social response or how social groups (family, friends, and society in general) influence consumers and the behavioral response or how personality, demographic origin, and lifestyle may determine purchase choices is also considered part of the consumer’s psychological aspects. Readers interested in the relation between emotions and food are directed to [4,5] for comprehensive reviews.

The physiological reactions toward products have recently become of interest for the consumer behavioral studies community. Several attempts have been made to measure physiological reactions accurately and, thus, predict a new product’s market performance: heart rate, body temperature, galvanic skin response (GSR), electroencephalography (EEG), visual attention, and facial expressions have been considered as potential hints to determine consumer preference towards a product. Still, recognition of physiological reactions elicited by food products is a novel discipline, and the proper algorithms to interpret them are yet to be developed [6].

Within this context, Viejo et al. evaluated in [7] the EEG, heart rate, temperature, and facial expressions of beer consumers. In [8], He et al. recorded facial expressions of participants exposed to orange and fish odors. Motoki et al. implemented an eye-tracking system to evaluate visual attention elicited by food images [9]. Leitch et al. measured responses to sweeteners in tea through a hedonic scale, an emotion term questionnaire, and facial expressions [10]. Danner et al. reported gauging changes in skin conductance level, skin temperature, heart rate, pulse, and facial expressions of people tasting different juice samples [11]. Similarly, other authors have conducted studies with smoked ham [6] and bitter solutions [12]. A common feature of the aforementioned projects is that they all relied on FaceReader [13], a general purpose and commercially available facial emotion recognition (FER) software.

Other approaches have explored brain activity due to food valuation. Kuhn et al. used functional magnetic resonance imaging (fMRI) to study the effects of viewing and trying chocolate products [14]. Motoki et al. reviewed in [15] the influence of food-extrinsic factors and the mechanisms behind their integration in the human brain.

FER has gained much attention in the field of sensory analysis. Two approaches can be identified in FER [16]: the continuous model and the categorical model. While the former postulates a wide spectrum of different emotions, the latter focuses on a discrete set of basic emotions.

In particular, the categorical model, proposed by Paul Ekman in [17], remains the most popular. Through specialized training, Ekman’s method allows identifying face emotions by analyzing certain facial muscular activations. Nevertheless, both manual and automated training processes require many hours, while the facial analysis takes around an hour for each minute of video [18]. This is why a good amount of research has been devoted to finding computer algorithms that are able to outperform the current evaluation of facial expressions.

Deep convolutional neural networks (CNNs) have obtained good results in real-world FER applications [19] besides being robust and reducing variations across faces [16]. Among the different works that rely on CNNs for FER are those of Cai et al. [19], who proposed a new loss function, which tries to maximize the differences between predicted classes in FER-applied CNNs. Zhao et al. implemented in [20] a 3D CNN architecture to learn features in facial images and optical flow sequences. Li et al. [21] used an attention mechanism in CNNs to classify facial expressions even in partially occluded faces by focusing on different regions of a facial image and weighing them according to the level of occlusion they exhibit and how much they contribute to the classification. Wang et al. [22] tried to improve recognition accuracy by focusing on combining multiple weighted regions of facial images. Liong et al. designed a shallow (only two layers) triple stream 3D CNN that was capable of extracting high level features, as well as micro-expressions through determination of optical flow features [23].

In addition to FER, novel types of analyses including recording of emotions and physiological changes elicited in consumers when trying new food products have contributed to achieving a more complete understanding of consumer responses [24].

In general, two different kinds of analyses have contributed to improve sensory evaluation: explicit and implicit.

Explicit analyses involve questionnaires that make use of verbal and non-verbal descriptor terms [25,26,27]. They have many advantages: they are easy to understand by consumers and relatively fast to decode. Some drawbacks are that results might be cognitively biased [27], and they do not record the consumer experience at the precise moment of tasting the product.

On the other hand, implicit methods measure FER and other physiological changes. For the latter, a comprehensive review that explains the response patterns for certain emotions can be found in [28]. Other implicit methods measure heart rate, skin conductance, skin temperature, and pupil dilation, among other physiological changes, and autonomic nervous system responses [25,26,28].

Though it has been reported that the perception of basic tastes is linked with specific facial movements (for example, sourness with the lips or bitterness with the eyes and forehead) [29], many different variables can affect both FER and physiological changes: how hungry a consumer feels, the type of food tested, and the time elapsed since the beginning of the test. Even in tests as short as 10 s, a consumer may exhibit several facial expressions [30]. Moreover, changes in facial expression are harder to determine when tasting food products, compared to smelling a perfume or watching a video [25], since the jaw movement produced by chewing and the occasional facial occlusion (because of the hand that takes the sample to the mouth) are often the cause of misreading in FER algorithms. These might be some of the reasons why similar studies seem inconclusive [7,31,32].

The present work aims to take a step towards a more reliable prediction of consumer acceptance by means of CNNs and other machine learning algorithms that interpret facial expressions and find potential correlations between biometric sensor measurements, facial analysis, and reported liking.

In this work, a self-developed FER system is introduced since not all studies based on commercial solutions have proven to be successful. Programming our own application provides more flexibility and allows exploring different methods and emotion models. Given that emotions are expressed multi-modally [33] and the fusion of information channels helps to improve predictions [34], we included biometric sensors in the analyses as well. The CNN presented in this paper is comprised of four channels, one for each quadrant of the facial image, as previous findings suggested that multiple networks performed better than an individual ones [16].

The rest of the paper is organized as follows: Section 2 presents the materials and methods used for the implementation of the proposed sensory evaluation system. Section 3 presents the results obtained, while Section 4 poses some discussion. Finally, Section 5 concludes the paper, summarizing the main contributions and giving future work perspectives.

## 2. Materials and Methods

### 2.1. Sensory Analysis

#### 2.1.1. Flavor and Odor Sample Description

For the experiments, the following ingredients and percentages were used to prepare sweet gums of five different flavors: glucose (36.5%, Deiman, El Paso, TX, USA), sugar (33.21%, Gelita, Lerma, Mexico), water (23.38%), unflavored gelatin (5.3%, Gelita, Lerma, Mexico), citric acid (1.28%, ENSIGN, Shandong, China), flavor (0.3%), and red color (0.03%, Deiman, USA). For two flavors (clam and cheese), maltodextrin was used instead of sugar.

**Figure 1:**
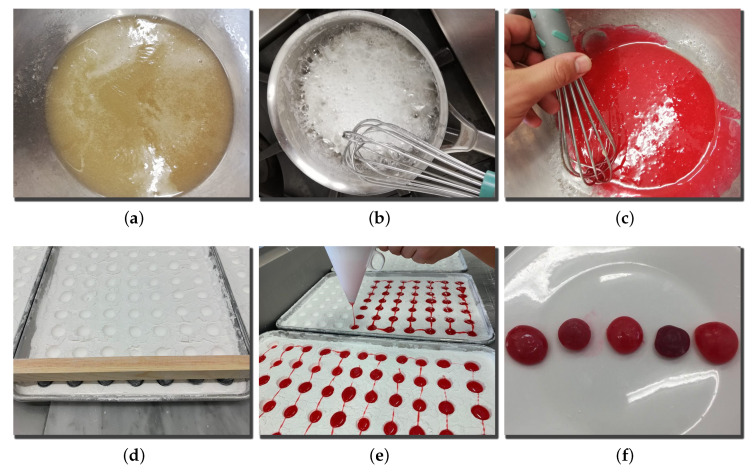
Sweet gum elaboration process: (**a**) gelatin in water, (**b**) sugar, water, and glucose mixture, (**c**) gelatin solution, color, flavor, and citric acid in the mixture, (**d**) bed of starch, (**e**) mixture in the bed of starch, and (**f**) resulting sweet gums.

The procedure for the elaboration of sweet gums is depicted in Figure 1 and is as follows:

1. Let unflavored gelatin dissolve in water (10.6 g/L) for 30 min.
2. Mix sugar and water (11.5 g/L) and heat at 70 °C; add glucose, and increase the temperature up to 108 °C.
3. Add the unflavored gelatin solution, color, and flavor to the mixture at 100 °C, as well as diluted citric acid (1.28 g/L).
4. Finally, cast the mixture in a bed of starch and let it rest for 18 h.

All the resulting sweet gums had similar appearances (Figure 1f) in order to keep volunteers from guessing the sample’s taste beforehand. Sweet gums can control the flavor release at the precise moment when the product is tasted, and therefore, the facial expression can be measured at the exact moment that the consumer receives the stimuli. These sweet gums’ flavors were determined beforehand in order to provide five different sensory stimuli. We used three flavors considered as pleasant: mint (Deiman, USA), pineapple (Deiman, USA), and strawberry (Deiman, USA), as well as two unpleasant ones: clam (Bell, Northbrook, IL, USA) and Gouda cheese (Bell, USA).

**Figure 2:**
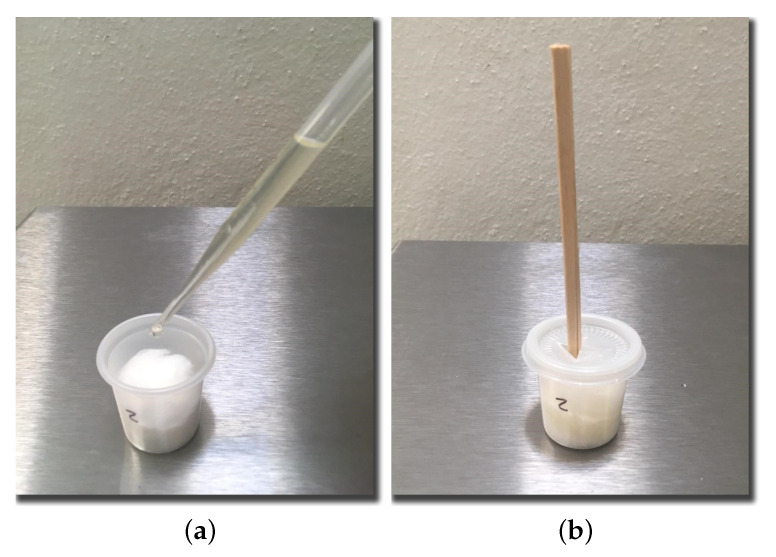
Odor sample: (**a**) Soaking cotton with solution. (**b**) Wooden stick in container.

We also prepared a set of odor samples by soaking pieces of cotton in different solutions within a sealed container. Test participants would use a small wooden stick to bring the substance closer to their nose (Figure 2). The odors used for this experiments were: pineapple (Ungerer, Lincoln Park, NJ, USA), mint (Deiman, Horizon City, TX, USA), vinegar (Ungerer, Lincoln Park, NJ, USA), Gouda cheese (Bell, Chicago, IL, USA), and smoke (Castells, Mexico city, Mexico).

#### 2.1.2. Participants and Setup

A group of 120 students, professors, and administrative staff from Universidad Panamericana (Mexico) volunteered for the test. The experiment was conducted in a Sensory Laboratory with a controlled illumination booth. The booth was equipped with a Kinect device, which integrated various sensors such as a color camera, an infrared light camera, and a depth sensor, all in a single device, thus eliminating the need to manage and synchronize multiple sources of information.

**Figure 3:**
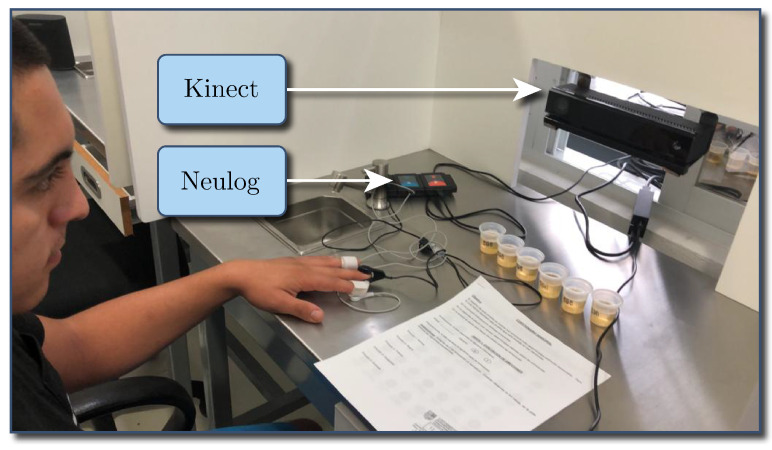
Booth setup.

For the present study, the Kinect device acquired the participants’ frontal facial images. During the test, a Neulog NUL-217 device was attached to each volunteer’s middle and ring fingers, as well as a Neulog NUL-208 attached to the index finger to measure both galvanic skin response (GSR) and cardiac pulse, respectively. The experimental setup is shown in Figure 3.

A small semaphore device was used to let each participant know the right moment to try each sample. This allowed a better synchronization between the reaction of the user and the records captured by the Kinect camera. All participants were instructed to try a sample at a specific moment while being filmed by the Kinect. After trying each sample, consumers had water and salted crackers to neutralize the flavors. Finally, the consumers were instructed to answer a sensory questionnaire.

#### 2.1.3. Questionnaire

A questionnaire comprised of a seven-point hedonic scale for each of the five samples of taste and odor was implemented. These questionnaires are commonly used in sensory science for testing acceptance of different types of food products.

The results obtained with the sensory questionnaires were compared to those obtained from the facial expressions using artificial intelligence methods.

### 2.2. System Architecture

**Figure 4:**
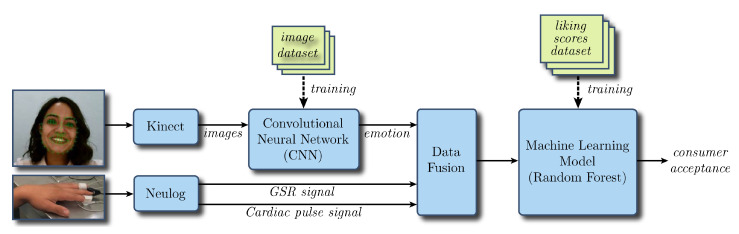
The sensory analysis system architecture. GSR, galvanic skin response.

Figure 4 shows the system’s architecture, depicting its main modules. Our sensory analysis system used three inputs: facial images, GSR signals, and cardiac pulse signals. As previously mentioned, facial images were acquired by a Kinect device, while the GRS and cardiac pulse signals via a Neulog device.

Facial images were analyzed by a previously trained CNN to determine the consumer’s emotion. The detected emotion together with the GSR and pulse signal values followed a statistical-based data fusion process. The result went to a machine learning model, which in turn predicted the consumer acceptance.

The machine learning model was based on the random forest classification method, and it used the consumer’s liking scores and the values resulting from the data fusion stage for training. Once trained, the liking scores were no longer needed. This approach targeted eliminating their use for predicting consumers’ acceptance. Having just an implicit method for measuring acceptance eliminated external factors and their influence on the results.

The following subsections will detail the system’s modules.

### 2.3. Neural Networks

**Figure 5:**
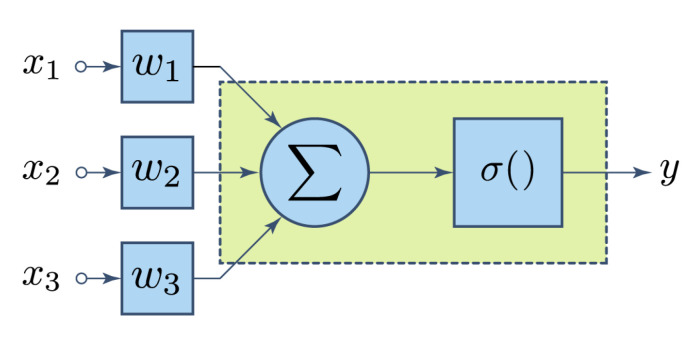
Schematic representation of a three-input perceptron.

A neural network is an interconnected assembly of processing elements whose functionality loosely resembles that of a neuron [35]. These processing elements are commonly known as perceptrons. Figure 5 shows their basic structure: a set of numerical inputs $x_{i}$ are weighted by a corresponding factor or weight $\omega_{i}$. Results are then added. Finally, an activation function σ is applied to the sum to yield the final result *y*. For any input vector x of length *n*, this functionality can be expressed by Equation (1):

(1) $$\begin{array}{c} y=\sigma (\sum_{i=1}^{n}w_{i}x_{i}) \end{array}$$

**Figure 6:**
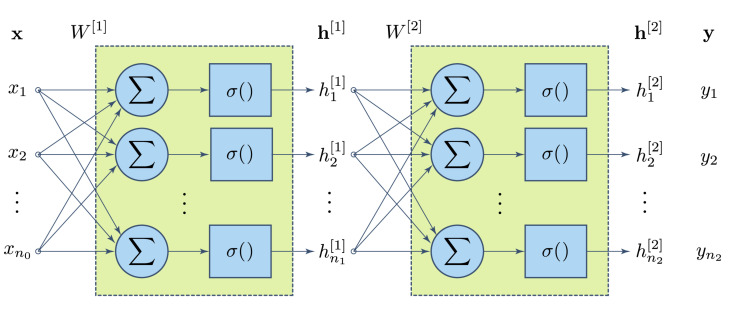
Two-layer neural network architecture. For the sake of simplicity and clarity, individual weights are not shown.

Many layers can be stacked together to configure larger neural networks for more complex classification tasks. Figure 6 depicts, for example, a two-layer neural network.

### 2.4. Facial Expression Datasets

Two different facial expression datasets: AffectNet [36] and CK+ [37], for training and testing the neural network, respectively, were used.

AffectNet contains more than 420,000 facial images classified among 11 discrete labels. However, in order to have a balanced training, only 3800 images associated with each of the next 10 labels were used: neutral, happy, sad, surprise, fear, disgust, anger, contempt, none, and uncertain. Images classified as non-face in the dataset were not included. We chose to use CK+ as the evaluation reference since it is a well-known dataset among researchers and is comprised of a much lower number of labeled images.

### 2.5. Image Preprocessing

**Figure 7:**
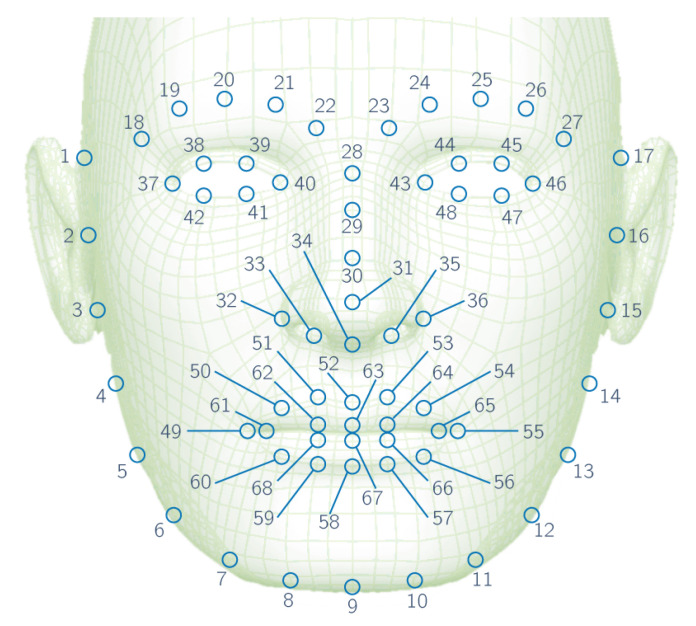
Numbered facial landmarks.

Images in the training dataset had some irregular characteristics that could not be handled by the neural network. Therefore, some preprocessing was required to assure that the network would receive only consistent information, namely:

1. Discard color information, converting RGB-coded images to gray scale in order to reduce their size and the processing time.
2. Detect all faces in the image, together with their bounding rectangles by applying an algorithm based on the histogram of gradients (HoG) [38].
3. Locate 68 landmarks on the first face detected using the Kazemi algorithm [39].
4. Rotate the image and landmarks to make the line between landmarks 40 and 43 horizontal, so that all processed faces are aligned (see Figure 7).
5. Divide facial image into four sections, specifically left and right sections for eyes and nose-mouth.
6. Flip right sections horizontally in order to feed left and right sections to the same network.
7. Equalize every section with contrast limited adaptive histogram equalization (CLAHE) [40].
8. Normalize pixel values in the range from (0,255) to (0,1).

These operations were performed through the Dlib [41] and OpenCV [42] libraries in the Python programming language, while those related to the neural network used the Keras library [43] for Deep Learning with the TensorFlow backend [44].

### 2.6. Network Architecture

**Figure 8:**
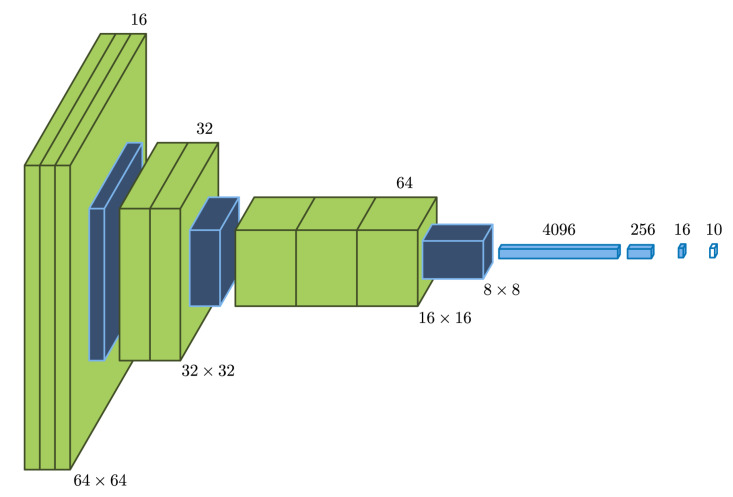
Neural network architecture for the first stage.

The first stage was composed of two networks that were trained differently, but shared the architecture shown in Figure 8: every image section (64×64 pixels) was fed through three convolutional layers and a max-pooling layer. Later, the other two similar filter blocks further reduced the 2D information to feed the other four dense row layers, the last of which produced a partial classification into 10 possible labels by means of a softmax transfer function. All previous transfer functions were rectified linear units (ReLUs).

Network A output a 10 number vector for left eye and flipped right eye sections, whereas Network B did the same for nose-mouth sections. The four resulting vectors would then work as a 40 number input for the second stage of the network. This stage encompassed two dense ReLU layers and a softmax output layer that yielded the final classification.

### 2.7. Network Training

Only 40,366 faces of the selected subset were fit for training since the face or landmark detection algorithms did not work properly for all cases. By flipping the right sections, a total of 80,672 facial images were available for training Networks A and B. Both networks were trained on 50 epochs with a batch size of 128. We used 20% of the training set for validation and a dropout rate of 0.4 in some layers to reduce the chance of overfitting.

Next, the networks processed all available faces to obtain 80,672 vectors of 40 elements, which were used as the training set for the second stage. In the corresponding training process, we applied the same parameters as the in first, except for the validation percentage, which was 15%.

### 2.8. Emotion Recognition

The trained network was fed with all the preprocessed images corresponding to 111 participants. An equal number of CSV files were obtained containing the following columns: image index, number of faces detected (or –1 if the algorithm was unable to find a face), image file name, and the probability of classification for all emotion labels mentioned in Section 2.4.

### 2.9. Data Fusion

**Figure 9:**
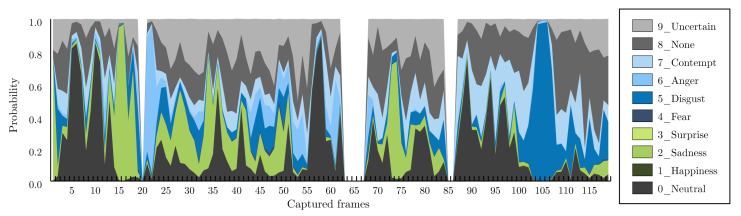
Example of detected emotion probabilities.

Each an experiment provided data from three different sources: (1) images that provided the response of nine facial expressions in a range (0,1), (2) GSR, and (3) pulse response. As shown in Figure 9, the sensor’s measurements were spread across a time series (measured in frames, which were recorded at a target rate of 30 frames per second), and several samples were stored for each experiment. To represent the sensors’ data, four statistical metrics were used: the average (avr), the standard deviation (std), the minimum (min), and the maximum (max) values. In sum, for each experiment, there were 44 features obtained from four statistical metrics of nine facial expressions, GSR, and pulse signals.

### 2.10. Acceptance Prediction

We used machine learning regression techniques to predict the acceptance that consumers assigned to each sample. For each experiment, we first extracted 44 input features, as previously explained, and one output: the level of acceptance assigned to the sample by the consumer. Each consumer evaluated 10 different samples.

The selected machine learning model for predicting the acceptance was random forest, as proposed by Breiman [45]. It was comprised of a set of random decision trees (30 for this work), each one created with a random subset of samples and features from the training dataset.

**Figure 10:**
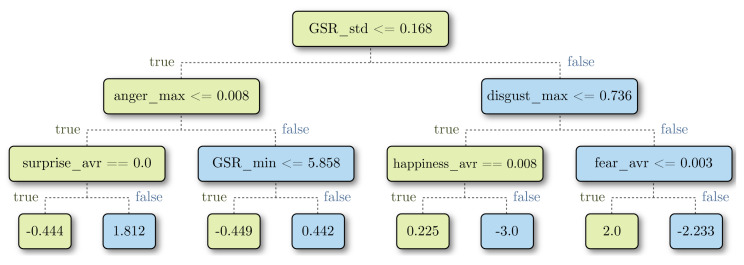
Decision tree example: to predict the consumer’s evaluation, questions need to be answered from top to bottom and following the path of the answers. At the end of the path, the last node contains the prediction of the consumer’s evaluation.

A decision tree is a prediction model based on a series of questions about features’ values (Figure 10). In a decision tree, data are separated into many dimensions by hyperplanes, which are determined as a result of the questions. The main idea is that samples with similar values tend to concentrate in the same region. We chose random forest because it measures and shows how much each feature contributes to the final model. Decision trees establish selection criteria (Figure 10) by trying to minimize the impurity of the data in each node. In this case, the impurity was calculated as the mean squared error (MSE), formally Equation (2).

(2) $$\begin{array}{c} MSE\left(\vec{y},\vec{\hat{y}}\right)=\frac{1}{n}\sum_{i=1}^{n}{\left(y_{i}-{\hat{y}}_{i}\right)}^{2}, \end{array}$$

where $\vec{y}$ and $\vec{\hat{y}}$ are the real and the predicted outputs (the reported acceptance values in the experiments), respectively, and *n* is the number of samples. When a classification rule is defined, the node data are split into two regions. Several features and values were tested, and the feature-value pair that minimized the impurity was selected as the classification rule.

Feature importance is proportional to the impurity reduction of all nodes related to that feature. The impurity reduction IR in each node *j* representing a rule can be calculated with Equation (3):

(3) $$\begin{array}{c} IR_{j}=w_{j}I_{j}-\left(w_{left}I_{left}+w_{right}I_{right}\right), \end{array}$$

where left and right represent the children nodes of node *j*, *I* represents the impurity of each node, and the weights *w* are the samples’ proportion in nodes, and they are calculated as the number of samples in the node divided by the total number of samples. Once the impurity reduction in all nodes is known, the importance of the feature *k*, $FI_{k}$, is calculated using Equation (4):

(4) $$\begin{array}{c} FI_{k}=\frac{\sum_{j\in N_{k}}IR_{j}}{\sum_{j\in N}IR_{j}}. \end{array}$$

where $N_{k}$ represents the set of all nodes that are split using variable *j* and *N* represents all the nodes in the decision tree.

Results were validated through a ten-fold cross-validation. This meant that the dataset was randomly divided into ten blocks. The model was later fitted ten times using nine blocks for training and one block for testing. The mean absolute error (MAE) was used for calculating the model error. Formally, Equation (5):

(5) $$\begin{array}{c} MAE\left(\vec{y},\vec{\hat{y}}\right)=\frac{1}{n}\sum_{i=1}^{n}\left|y_{i}-{\hat{y}}_{i}\right| \end{array}$$

where $\vec{y}$ and $\vec{\hat{y}}$ are the real and the predicted outputs, respectively. The MAE was calculated each time the model was fitted and tested. The final results were the average of all runs. We chose to present these results with MAE as opposed to the MSE used for fitting the model, since it was easier for interpretation.

## 3. Results

**Figure 11:**
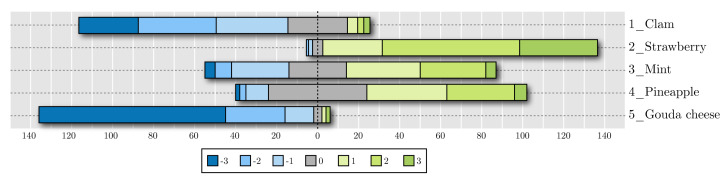
Acceptance results for taste evaluations.

**Figure 12:**
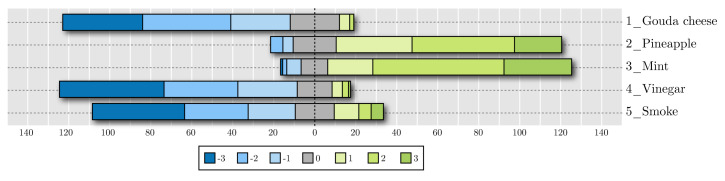
Acceptance results for smell evaluations.

Figure 11 and Figure 12 show the cumulative results for hedonic scales in the taste and smell evaluations, respectively. The bars, centered on zero, represent how many participants rated each smell or flavor. These figures show the results of the questionnaire with Likert scales ranging from –3 (the most disliked) to three (the most liked).

Strawberry was the most liked flavor, and Gouda cheese seemed to elicit the worst reaction, since its most common score was −3 and almost the whole bar lied on the left side of the chart. Clam obtained a negative overall score as well. As for smell tests, pineapple and mint had a good acceptance, while Gouda cheese, vinegar, and smoke did not. There seemed to be a good contrast between reported acceptance of liked and disliked samples.

**Figure 13:**
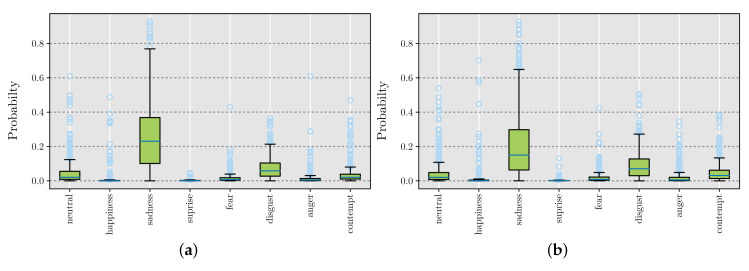
Recognized emotions: (**a**) taste and (**b**) smell experiments.

Figure 13 shows the emotions that were recognized during the taste (Figure 13a) and smell (Figure 13a) experiments. The boxplots represent the average probability value of each emotion for all consumers during the five experiments. It can be observed that sadness was the emotion that mostly appeared during the execution of the experiments followed by disgust.

Our results agreed with those of He et al. [30]. In this study, He and coworkers measured the changes of facial expression for the same, similar, and different taste conditions. They concluded that the pleasantness of consuming a food product diminished very rapidly; therefore, in this finding, the expressions of sadness and angry were found to be predominant. Additionally, we noted that the expressions of sadness and disgust were probably due to consumers arriving nervous and with uncertain expectations for the experiment, then, once in the test, with their important concentration for perceiving all tastes and odors.

**Figure 14:**
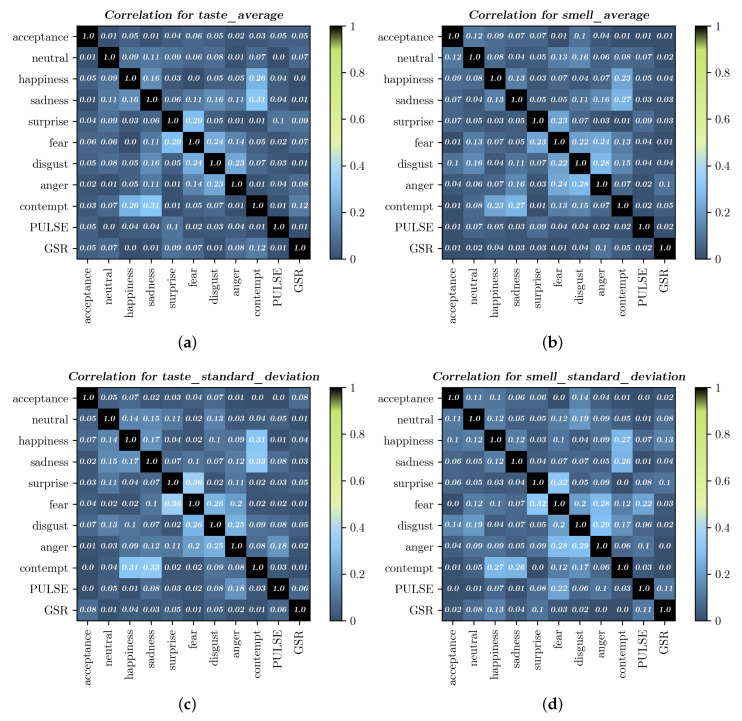
Correlation matrix of facial emotion recognition (FER), sensor responses, and consumer acceptance: (**a**,**c**) display the correlation matrices of taste experiments, while (**b**,**d**) show the correlation matrices of smell experiments. Cells contain correlations between column and row features.

Figure 14 shows the correlation matrices of FER, sensor responses, and consumer acceptance in the different experiments. Values on the matrices were calculated using the absolute Pearson’s correlation coefficient. No strong correlation between consumer acceptance and other features was found. However, the features with higher correlations with consumer acceptance were the following: in Figure 14a: fear, happiness, disgust, pulse, and GSR; in Figure 14b: neutral and happiness; in Figure 14c: GSR, happiness, and disgust; in Figure 14d: disgust and neutral.

We interpreted that fear, happiness, disgust, neutral, pulse, and GSR were the features with higher correlation with consumer acceptance. However, these correlations were too weak. Fear was the most difficult expression to recognize accurately with static images [46]. Fear was usually miscategorized together with surprise by both humans and FER models [46,47].

Table 1 shows the MAE of our regression model, as described in Equation (5), which predicted sample acceptance based on FER and the sensors’ detected responses. The first column describes the type of data used to train the random forest. The model obtained the best prediction when trained with the GSR measurements alone. These results were similar to those obtained in previous works [48,49].

**Figure 15:**
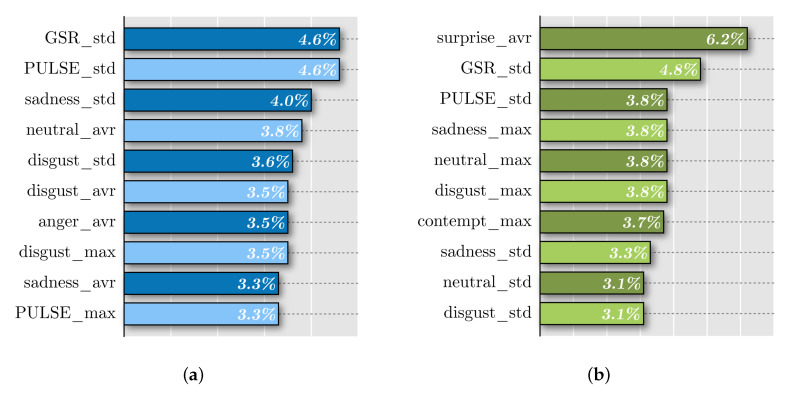
Feature importance: (**a**) taste and (**b**) smell regression models.

As mentioned in Section 2.10, our random forest model rated the importance of each feature in predicting acceptance. The ten most important features for each set of experiments are shown in Figure 15.

The standard deviations of pulse and GSR samples showed up as the most relevant variables to take into account when predicting acceptance. The average of surprise measurements emerged as the top variable in the left column. However, it was absent in the right one. This could be explained by the fact that the sense of smell may elicit greater emotions than taste. Nevertheless, emotion measurements were very similar in both smell and taste experiments (Figure 13). This suggested that the GSR and pulse sensors were better predictors than the CNN array.

A box plot was obtained for every sample. Note that no relevant variance could be found among them. For this reason, only the average recorded values for every detectable emotion were included, as shown in Figure 13.

**Figure 16:**
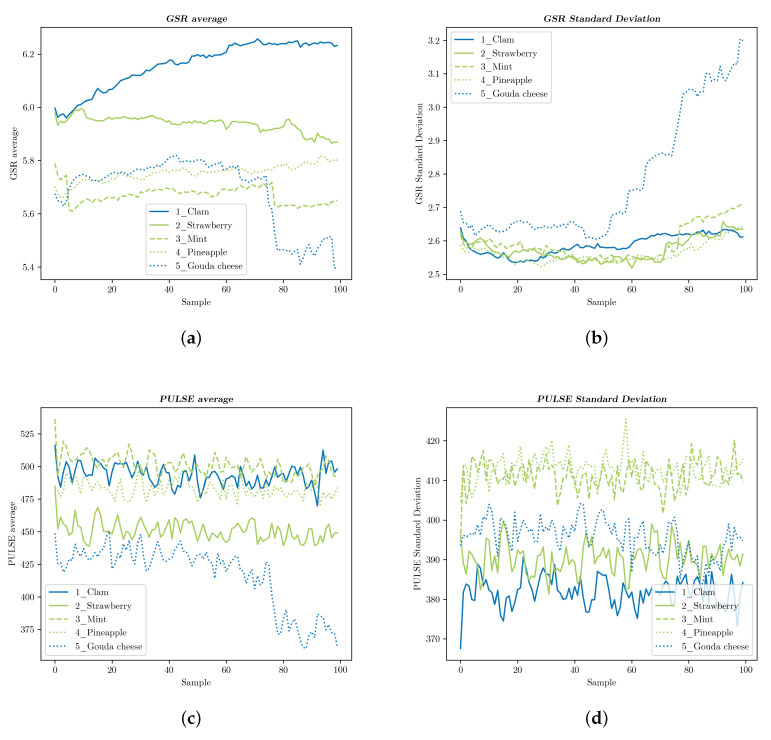
GSR and pulse measurements for the taste tests: (**a**) The participants’ GSR average values and (**b**) standard deviation. (**c**) The participants’ pulse average values and (**d**) standard deviation.

**Figure 17:**
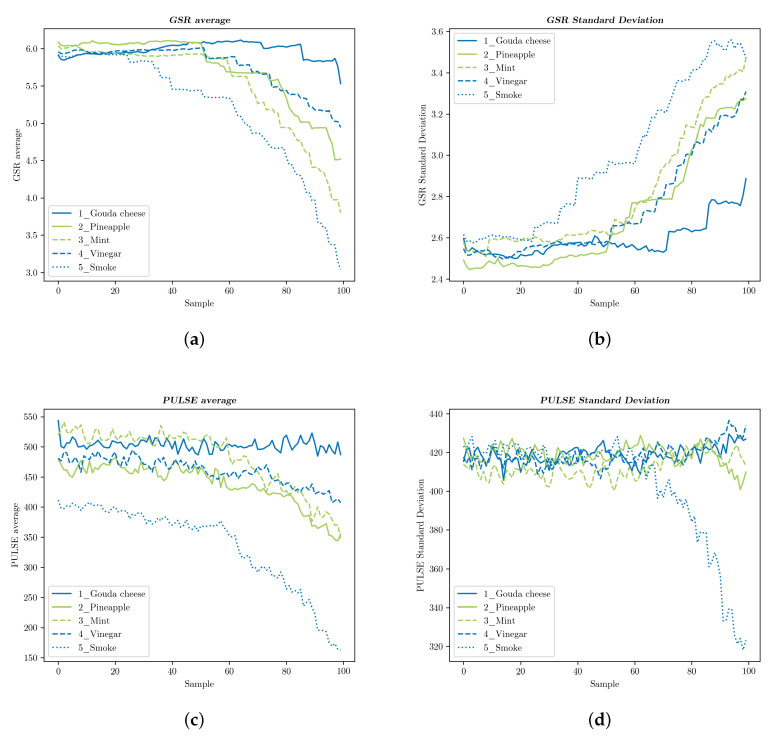
GSR and pulse measurements for the smell tests. (**a**) The participants’ GSR average values and (**b**) standard deviation. (**c**) The participants’ pulse average values and (**d**) standard deviation.

We calculated the average value and standard deviation of all participants’ GSR and pulse, once for each of the 100 samples, which were obtained at a rate of eight per second in arbitrary units, as provided by the sensors. Figure 16 and Figure 17 display these results: blue graphs represent flavors and smells reported as disliked, whereas those related with liked samples are shown in green. In Figure 16, two flavors seemed to be producing relevant changes in the sensors’ measurements, both of which were clearly reported as disliked: the GSR average of samples slowly rose for 1_Clamand abruptly fell for 5_Gouda cheese, while the standard deviation of the latter distinguished itself from the remaining graphs because of its steep increase. No disparity was shown between the taste samples reported as liked: 2_Strawberry, 3_Mint, and 4_Pineapple. Furthermore, they remained almost constant over time.

Figure 17 shows a similar pattern for two curves associated with disliked samples: the value of the GSR average in 5_Smoke plunged, while it stayed above the others for 1_Gouda cheese, with the resulting changes in the standard deviation. 4_Vinegar, on the other hand, followed the same trend as those samples that scored high on liking: 2_Pineapple and 3_Mint. The smell of vinegar might induce weaker reactions than the reported liking scores suggested.

Average pulse readings revealed again characteristic curves for 1 and 5, but only the standard deviation of 5_Smoke showed a clear distinction. Once again, and except for 4_Vinegar, smells reported as disliked were separated from the rest in some way, which suggested that these features were indicative of strong (or at least detectable) emotional reactions.

## 4. Discussion

Automatic facial emotion recognition (FER) by itself is not a problem with a single straightforward solution; still, many variables must be considered. To this day, the best reference for rating observed emotions is human assessment, which is still prone to misclassifications [33] even with proper training [50], because emotion recognition is context-dependent [33,47] and thus relies heavily on a cognitional understanding of the situation in which an emotion is produced. On top of that, in experiments similar to ours [6,32], consumers showed almost no facial gestures, even for acute stimuli [2], and some expressions proposed as innate were rarely observed [51]. All of this might explain why our FER approach, as well as similar ones constantly detected just a couple of emotions.

However, Bredie et al. [2] and Crist et al. [12] were successful in evoking expressions of disgust with highly concentrated solutions of caffeine, citric acid, and sodium chloride. The use of analogous stimuli should be taken into account for future work as it might help CNNs detect facial expressions more clearly. Gunaratne et al. [24] reported expressions of sadness positively associated with the tasting of salty chocolate. This may provide a hint to find out why the proposed FER system yielded a classification of sadness so often.

Finally, the correlations found between emotions and hedonic scores were very low, just as those reported by Litch [10]. It could be therefore concluded that the connection between food consumption and experienced emotions, as well as the connection between real and FER reported emotions were much weaker than expected, at least when appraised in this manner.

On the other hand, FER for food product assessment has been the subject of very few studies, and the required algorithms are yet to be developed [6]. Nevertheless, our results, as well as those obtained by Samant et al. [26] suggested that GSR measurements were more reliable for pointing out emotional reactions. Though further research is still necessary to confirm that liking can be associated with an increased heart rate as stated by De Wijk et al. [52], their assertion of emotional intensity being associated with a reduced heart rate could be confirmed to some extent by the graphs shown in Figure 17. Further studies should not leave out this type of sensor in order to validate our present results.

## 5. Conclusions

Measuring physiological signals and images to determine consumer acceptance as part of, or in addition to, other sensory tests is gaining attention in sensory science. In this context, this paper presented a novel automatic sensory analysis system, which aimed to predict consumers’ acceptance when trying new food products.

The system encompassed facial emotion recognition (FER), galvanic skin response (GSR), and cardiac pulse together with liking reports. A novel artificial intelligence based approach for data fusion of consumers’ facial images and biometric signals was proposed to determine the preference elicited by food products. Two input channels were used and compared: taste and smell.

The experiments conducted to validate this approach involved the participation of a group of 120 voluntary subjects. The significant amount of data obtained was processed using machine learning techniques such as neural networks, statistical metrics, and decision trees.

Results showed that FER alone was not sufficient to determine consumers’ acceptance. In line with previously reported works, the facial expressions of sadness and disgust were constantly detected probably due to consumers feeling nervous, anxious, or simply concentrating during the experiment. However, when correlated with GSR and pulse signals, acceptance prediction could be improved. Our experiments showed that GSR was the most relevant variable to take into account when predicting product acceptance. Cardiac pulse, to a lesser extent, could be confirmed to be related to emotional intensity elicited by food products.

The proposed approach was proven to be efficient at processing and correlating different kinds of input signals and big amounts of data. Future studies will investigate the use of EEG signals as an additional biometric input to the model and the use of intense flavors and smells (such as citric acids, perfumes, and hydrogen sulfide) to induce clear facial expressions.

## Figures and Tables

**Table 1:** Mean absolute error (MAE) for the regression model.

Data	Taste	Smell
sensors + emotions	1.8216	1.8593
emotions only	1.8408	1.8273
sensors only	1.7896	1.8493
GSR only	1.7649	1.7817
pulse only	1.8173	1.9655
